# Type 2 diabetes mellitus is associated with an imbalance in circulating endothelial and smooth muscle progenitor cell numbers

**DOI:** 10.1007/s00125-012-2590-5

**Published:** 2012-06-01

**Authors:** J. van Ark, J. Moser, C. P. H. Lexis, F. Bekkema, I. Pop, I. C. C. van der Horst, C. J. Zeebregts, H. van Goor, B. H. R. Wolffenbuttel, J. L. Hillebrands

**Affiliations:** 1 Department of Pathology & Medical Biology–Pathology, University of Groningen, University Medical Center Groningen, Hanzeplein 1, PO Box 30.001, Groningen, the Netherlands; 2 Department of Cardiology, University of Groningen, University Medical Center Groningen, Groningen, the Netherlands; 3 Department of Surgery–Vascular Surgery, University of Groningen, University Medical Center Groningen, Groningen, the Netherlands; 4 Department of Endocrinology, University of Groningen, University Medical Center Groningen, Groningen, the Netherlands

**Keywords:** Atherosclerosis, Macrovascular disease, Stem cells, Type 2 diabetes mellitus

## Abstract

**Aims/hypothesis:**

Individuals with type 2 diabetes mellitus have increased rates of macrovascular disease (MVD). Endothelial progenitor cells (EPCs), circulating angiogenic cells (CACs) and smooth muscle progenitor cells (SMPCs) are suggested to play a role in the pathogenesis of MVD. The relationship between vasoregenerative EPCs or CACs and damaging SMPCs and the development of accelerated MVD in diabetes is still unknown. We tried to elucidate whether EPC, CAC and SMPC numbers and differentiation capacities in vitro differ in patients with and without diabetes or MVD.

**Methods:**

Peripheral blood was obtained from insdividuals with and without diabetes and MVD (coronary or peripheral artery disease). EPC and SMPC numbers were determined with flow cytometry. Furthermore, CAC and SMPC numbers were quantified after in vitro culture. Their in vitro differentiation capacity was investigated with real-time RT-PCR and quantitative immunofluorescence.

**Results:**

In diabetic patients both EPC and CAC levels were reduced (1.3-fold [*p* < 0.05] and 1.5-fold [*p* < 0.05], respectively). CAC outgrowth from diabetic patients with MVD was reduced 1.5-fold compared with diabetic patients without MVD (*p* < 0.05). SMPC levels were similar between diabetic patients and healthy controls. The CAC/SMPC ratio of in vitro cultured progenitor cells was reduced 2.3-fold in samples from diabetic patients (*p* < 0.001). The differentiation capacity of CACs and SMPCs in vitro remained similar independently of diabetes or MVD.

**Conclusions/interpretation:**

The ratio between EPCs or CACs and SMPCs is disturbed in type 2 diabetes in favour of SMPCs. This may translate into reduced vascular repair capacity, thereby promoting MVD in type 2 diabetes.

**Electronic supplementary material:**

The online version of this article (doi:10.1007/s00125-012-2590-5) contains peer-reviewed but unedited supplementary material, which is available to authorised users.

## Introduction

Type 2 diabetes mellitus is a complex disease that is associated with an increased risk of developing macrovascular disease (MVD). Patients with diabetes have a two- to fourfold increased risk of developing coronary artery disease (CAD) and peripheral artery disease (PAD) compared with non-diabetic individuals [1]. MVD is mainly the result of atherosclerosis. The mechanism(s) behind the accelerated development of atherosclerosis in diabetes is still not fully understood [2] and cannot be fully explained by the combined effect of established risk factors such as hypertension and dyslipidaemia.

There is increasing evidence that circulating vascular progenitor cells play a role in vascular homeostasis and may contribute to the development of MVD [3]. At least two types of vascular progenitor cells have been identified: endothelial progenitor cells (EPCs) and smooth muscle progenitor cells (SMPCs). EPCs contribute to endothelial regeneration and postnatal neovascularisation [4, 5] by differentiating into mature endothelial cells and incorporating into new vessels and/or by secreting pro-angiogenic growth factors that enhance vascularisation mediated by resident endothelial cells in a paracrine fashion [6–8]. The CD34^+^ population of circulating cells has been shown to contain EPCs, and a subpopulation of CD34^+^ cells co-expressing kinase domain receptor (KDR; also known as vascular endothelial growth factor receptor 2 [VEGFR-2]) has been shown to be an independent predictor of cardiovascular events in patients with CAD [9, 10]. In addition, cells with vasoregenerative potential can be cultured from peripheral blood mononuclear cells (PBMCs) using culture conditions favouring endothelial differentiation [11]. Cells grown under these conditions were formerly termed ‘early EPCs’, but are currently referred to as ‘circulating angiogenic cells’ (CACs). It has been shown that these cells are of myeloid origin and acquire endothelial-like characteristics in vitro. While these cells have not been shown to differentiate into mature endothelial cells they can contribute to vascular repair [12].

It is now well established that several cardiovascular risk factors, including diabetes, are negatively correlated with EPC frequency in the circulation [13, 14]. The increased incidence of atherosclerosis in diabetes has previously been correlated with a reduction in the number, and changes in the function of, EPCs [15]. In addition to EPCs it has been shown that there is also a population of bone-marrow-derived circulating vascular progenitor cells that have the capacity to differentiate into smooth muscle cells (SMCs) [16]. Animal and human studies indicated that these so-called SMPCs may contribute to the development of atherosclerosis by differentiating into mature SMCs or by the secretion of pro-inflammatory growth factors and cytokines that promote atherosclerotic plaque growth in a paracrine fashion [17–19]. A specific subset of circulating monocytes expressing CD14 and CD105 has been shown to contain cells with SMPC characteristics. SMPCs can also be obtained in vitro by culturing PBMCs using selective culture media [20, 21]. It has previously been shown that patients with CAD have increased numbers of CD14^+^CD105^+^ SMPCs [20]. Furthermore, Nguyen et al showed that SMPC levels were increased in the peripheral blood of patients with type 1 diabetes [22]. In addition, Westerweel et al observed a disturbed balance between circulating EPCs/CACs and SMPCs (in favour of the latter) in patients with end-stage renal disease (ESRD) who, like diabetic patients, are at increased risk of developing cardiovascular disease [21].

As yet, it is unknown whether SMPC levels are affected in type 2 diabetes. Therefore, we test here the hypothesis that in type 2 diabetes there is an imbalance between the number of circulating EPCs or CACs and SMPCs that favours increased differentiation of SMPCs, coinciding with reduced EPC/CAC-mediated repair capacity. Such a disturbed balance may contribute to increased risk of developing MVD in patients with diabetes. To this end, we assessed the number and phenotype of circulating EPCs and SMPCs using flow cytometry (FACS analysis) in individuals with and without type 2 diabetes and with or without MVD. To determine CAC and SMPC differentiation capacity towards endothelial and smooth muscle cells, respectively, in vitro cell culture was performed followed by quantification and differentiation analyses using quantitative immunofluorescence and gene expression analysis.

## Methods

### Individuals

Patients with type 2 diabetes and non-diabetic individuals with and without MVD were recruited and allocated into one of the following six groups: (1) diabetes, no MVD (*n* = 16); (2) diabetes with CAD (*n* = 15); (3) diabetes with PAD (*n* = 20); (4) no diabetes, no MVD (i.e. healthy controls, *n* = 19); (5) no diabetes with CAD (*n* = 16); and (6) no diabetes with PAD (*n* = 20). Type 2 diabetes mellitus was diagnosed using blood glucose cut-off values as defined by WHO. Non-diabetic patients with CAD or PAD were recruited from the UMCG cardiology and vascular surgery outpatient clinics, respectively. Diabetes patients with and without CAD or PAD were recruited from the UMCG Diabetes Center. Healthy volunteers were recruited from the general population. Samples, 50 ml, of peripheral blood were obtained by venipuncture and collected in EDTA vacutainers and 10 ml blood was collected in coagulation tubes (BD Biosciences, Franklin Lakes, NJ, USA). A more detailed description of patient selection criteria is provided in the electronic supplementary material (ESM; methods, section 1.1). Written informed consent was obtained from all study participants. The study protocol was approved by the local ethics committee of the University Medical Center Groningen (METc: 2008/335).

### Quantification of CPCs by flow cytometry

Progenitor cell frequencies were analysed with FACS in freshly collected whole blood samples. The total white blood cell (WBC) count in whole blood samples was determined with a Poch-100i haematology analyser (Sysmex Nederland, Etten-Leur, the Netherlands) according to the manufacturer’s instructions. EPCs were detected using antibodies directed against CD34 and KDR. SMPCs were defined by the dual expression of CD14 and CD105. For more a more detailed protocol on flow cytometry methods, please refer to the ESM (methods, section 1.2).

### CAC and SMPC culture

For CAC and SMPC quantification and characterisation in vitro, peripheral blood mononuclear cells were isolated and seeded in fibronectin-coated chamber slides. Pro-angiogenic and profibrotic culture media were used to obtain outgrowth of CACs and SMPCs, respectively. Details on progenitor cell culture methods are described in the ESM (methods, section 1.3).

### Characterisation of cultured progenitor cells

Immunofluorescence for differentiation markers for endothelial cells (ECs; endothelial nitric oxide synthase [eNOS] and KDR) and SMCs (α-smooth muscle actin [α-SMA] and collagen type 1) was performed to characterise the differentiation potential of in vitro cultured CACs and SMPCs. Details on immunofluorescent staining methods are described in the ESM (methods, section 1.4).

### Quantitative immunofluorescence TissueFAXS analysis

Immunofluorescent staining of CACs and SMPCs cultured in vitro were analysed with the TissueFAXS system (Tissuegnostics, Vienna, Austria). To quantify the number of adhering cells in a chamber slide well the number of nuclei was quantified based on DAPI staining. Subsequently, the staining intensity of specific antibodies was quantified based on Cy3 signal intensity. Details on TissueFAXS analysis methods are described in the ESM (methods, section 1.5).

### Gene expression profile of in vitro cultured CACs and SMPCs

Gene expression analysis was performed using the Taqman Low Density Array platform. Details on gene expression analyses are described in the ESM (methods, section 1.6).

### Statistical analysis

Data were analysed using Predictive Analytics SoftWare (PASW, version 18.0.3; IBM, Armonk, NY, USA) and GraphPad Prism software (version 5; La Jolla, CA, USA). Data are given as mean ± SEM. Dichotomous patient characteristics are expressed as percentages and compared with the *χ* ^2^ test. When comparing two groups the unpaired two-tailed Student’s *t* test was used. When comparing three or more groups ANOVA with the Bonferroni post hoc test was used. Differences were considered significant at *p* < 0.05.

## Results

### Patient characteristics

Patient characteristics are presented in Table 1. Our aim was to include patients with similar ages and sex ratios across all groups. While there were no significant differences in sex ratios across the groups we found that diabetic patients with MVD were, on average, slightly older than the patients from other groups. As anticipated, diabetic patients had significantly higher HbA_1c_ values compared with non-diabetic individuals (*p* < 0.001). Within the different subgroups of diabetic patients (with and without MVD), no differences in glycaemic control were observed. There was also no significant difference in diabetes duration between these groups.

**Table 1:** Patient characteristics

Characteristic	Type 2 diabetes without MVD (*n* = 16)	Type 2 diabetes with PAD (*n* = 20)	Type 2 diabetes with CAD (*n* = 15)	Healthy control (*n* = 19)	Non-type 2 diabetes with PAD (*n* = 20)	Non-type 2 diabetes with CAD (*n* = 16)	*p* value
Demographics							
Age (years)	58.6 ± 2.6	67.6 ± 1.6^a^	66 ± 1.7^b^	54.6 ± 1.0	59.6 ± 1.7	57.1 ± 2.4	<0.001
Sex (% male)	7 (44)	11 (55)	7 (47)	11 (58)	15 (75)	11 (69)	NS
Body mass index (kg/m^2^)	32.9 ± 1.6^c^	31.6 ± 1.9^c^	31.2 ± 2.0^c^	25.2 ± 0.7	23.2 ± 0.7	27.0 ± 1.0	<0.001
Type 2 diabetes duration (years)	17.5 ± 2.2	12.1 ± 1.9	15.1 ± 2.0				NS
Smoking (%)	5 (31)	4 (20)	2 (13)	3 (16)	13 (65)^j^	9 (56)	<0.01
Hypertension (%)	13 (81)	16 (80)	11 (73)	3 (16)^j^	13 (65)	9 (56)	<0.001
Metabolic variables							
WBC count (10^6^/ml)	8.1 ± 1.0	8.9 ± 0.7	6,3 ± 0,4	5.9 ± 0.4^d^	8.2 ± 0.5	6.5 ± 0.6	<0.01
Glucose (mmol/l)	6.4 ± 0.4	8.6 ± 1.0^e^	8.4 ± 0.8^e^	5.5 ± 0.2	5.5 ± 0.4	–^g^	<0.01
HbA_1c_ (%)	8.0 ± 0.4^f^	7.3 ± 0.3^f^	7.7 ± 0.3^f^	5.7 ± 0.1	6.0 ± 0.2	5.7 ± 0.1	<0.001
HbA_1c_ (mmol/mol)	64 ± 5^f^	56 ± 3^f^	61 ± 4^f^	39 ± 1	42 ± 1.9	39 ± 1	<0.001
Cholesterol (mmol/l)	4.1 ± 0.2	4.2 ± 0.2	4.4 ± 0.3	5.6 ± 0.2^i^	4.7 ± 0.4	4.4 ± 0.3	<0.01
Triacylglycerol (mmol/l)	1.7 ± 0.2	1.8 ± 0.1	2.5 ± 0.6	1.6 ± 0.2	1.9 ± 0.3	1.8 ± 0.3	NS
HDL-cholesterol (mmol/l)	1.3 ± 0.1	1.5 ± 0.2	1.3 ± 0.2	1.7 ± 0.1	1.3 ± 0.1	1.2 ± 0.1	NS
LDL-cholesterol (mmol/l)	2.3 ± 0.1	2.2 ± 0.2	2.5 ± 0.3	3.4 ± 0.2^j^	2.7 ± 0.3	2.7 ± 0.2	<0.01
Creatinine (μmol/l)	67.0 ± 4	74.2 ± 5.3	76.4 ± 5.4	77.7 ± 3.7	77.8 ± 5.0	79.7 ± 3.4	NS
Medication							
Insulin (%)	14 (88)	10 (50)	12 (80)	–	–	–	NS
Oral glucose-lowering agents (%)	2 (13)	4 (20)	1 (7)	–	–	–	NS
Metformin (%)	7 (44)	14 (70)	9 (60)	–	–	–	NS
Statins (%)	11 (69)	15 (75)	13 (87)	2 (11)^j^	16 (80)	16 (100)	<0.001
ACE inhibitors (%)	6 (38)	8 (40)	7 (47)	1 (5)^j^	10 (50)	12 (75)^j^	<0.001
Angiotensin II inhibitor (%)	7 (44)^d^	4 (20)	3 (20)	0 (0)	3 (15)	2 (13)	<0.05
Beta blockers (%)	3 (19)	7 (35)	11 (73)^j^	1 (5)^j^	3 (15)	15 (94)^j^	<0.001
Calcium antagonist (%)	5 (31)	6 (30)	8 (53)^j^	0 (0)^j^	4 (20)	1 (6)	<0.01
Diuretics (%)	9 (56)	7 (35)	8 (53)	1 (5)^j^	4 (20)	4 (25)	<0.05
Antiaggregants (%)	4 (25)	11 (55)	13 (87)	0 (0)^j^	14 (70)	15 (94)^j^	<0.001
Anticoagulants (%)	1 (6)	4 (20)	0 (0)	0 (0)	2 (10)	1 (6)	NS

Data are presented as mean ± SEM

Statistically significant with ANOVA compared with:

^a^Type 2 diabetic, non-type 2 diabetic with CAD, non-type 2 diabetic with PAD, and healthy

^b^Non-type 2 diabetic with CAD, and healthy

^c^Non-type 2 diabetic with PAD, and healthy

^d^Type 2 diabetic with PAD

^e^Healthy and non-type 2 diabetic with PAD

^f^All non-type 2 diabetic groups

^g^Measurement not available

Statistically significant with ANOVA compared with:

^h^Type 2 diabetic, type 2 diabetic with PAD, type 2 diabetic with CAD and non-type 2 diabetic with CAD

^i^Type 2 diabetic, type 2 diabetic with PAD and type 2 diabetic with CAD

^j^Statistically significant with *χ* ^2^ test

### Frequency of circulating CD34^+^ and CD34^+^KDR^+^ progenitor cells is reduced in type 2 diabetes

**Fig. 1:**
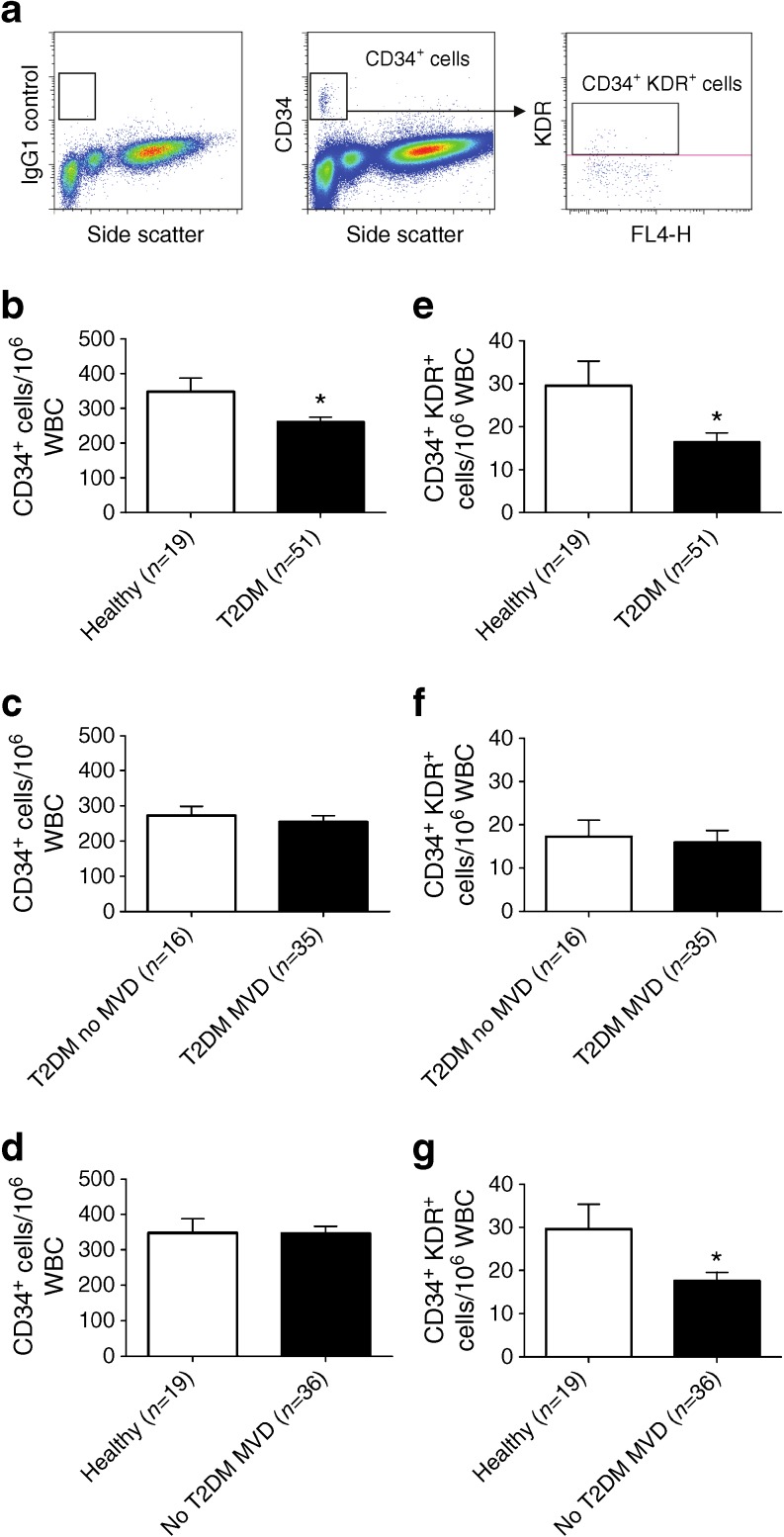
Circulating EPC levels are reduced in type 2 diabetes. (**a**) Representative FACS plots and gating profile set on the basis of the isotype control sample as used for the quantification of CD34^+^ cells and CD34^+^KDR^+^ cells. (**b**) CD34^+^ cell levels were 1.3-fold lower in type 2 diabetic patients compared with healthy controls. (**c**) In patients with type 2 diabetes, similar levels of circulating CD34^+^ cells were observed in patients with and without MVD. (**d**) In non-diabetic individuals with MVD the total number of CD34^+^ cells was similar to that in healthy controls. (**e**) CD34^+^KDR^+^ cell levels were 1.7-fold lower in type 2 diabetic patients compared with healthy controls. (**f**) In type 2 diabetes, similar levels of circulating CD34^+^KDR^+^ cells were observed in patients with and without MVD. (**g**) In non-diabetic individuals with MVD the number of CD34^+^KDR^+^ cells was significantly reduced compared with healthy controls. Data are expressed as mean values ± SEM; *p < 0.05. T2DM, type 2 diabetes mellitus

Circulating EPCs were enumerated using FACS analysis and were identified as cells with a low side scatter expressing CD34. Within the CD34^+^ cells, a subpopulation of CD34^+^KDR^+^ cells could be identified (Fig. 1a). To determine the relative frequency of EPCs compared with other circulating cells in the peripheral blood, EPC counts were expressed as the number of cells per 10^6^ WBCs. The frequency of CD34^+^ cells was significantly reduced (1.3-fold reduction) in diabetic patients compared with healthy controls (Fig. 1b, *p* < 0.05). Within patients with diabetes there was no difference in CD34^+^ cell frequency between diabetic patients with or without MVD (Fig. 1c). These results suggest that the presence of diabetes is responsible for the reduction in CD34^+^ cell levels without being influenced by the presence of PAD or CAD in these diabetic patients. This is supported by the observation that in non-diabetic individuals with MVD (CAD or PAD) the numbers of CD34^+^ cells were similar to numbers in healthy controls (Fig. 1d). In addition to the total CD34^+^ cell population, the numbers of circulating CD34^+^KDR^+^ cells were determined. Circulating CD34^+^KDR^+^ cell levels were significantly reduced (1.7-fold reduction) in diabetic patients compared with healthy controls (Fig. 1e, *p* < 0.05). Similar to the total number of CD34^+^ cells, there was no difference in the frequency of CD34^+^KDR^+^ cells observed between diabetic patients with and without MVD (Fig. 1f). In contrast to the total CD34^+^ cell population, in non-diabetic patients with MVD (PAD or CAD), the CD34^+^KDR^+^ progenitor cell subset was reduced to the same extent (1.7-fold reduction) as observed in diabetes when compared with healthy controls (Fig. 1g; *p* < 0.05).

### Frequency of circulating CD14^+^CD105^+^ SMPCs is increased in PAD

**Fig. 2:**
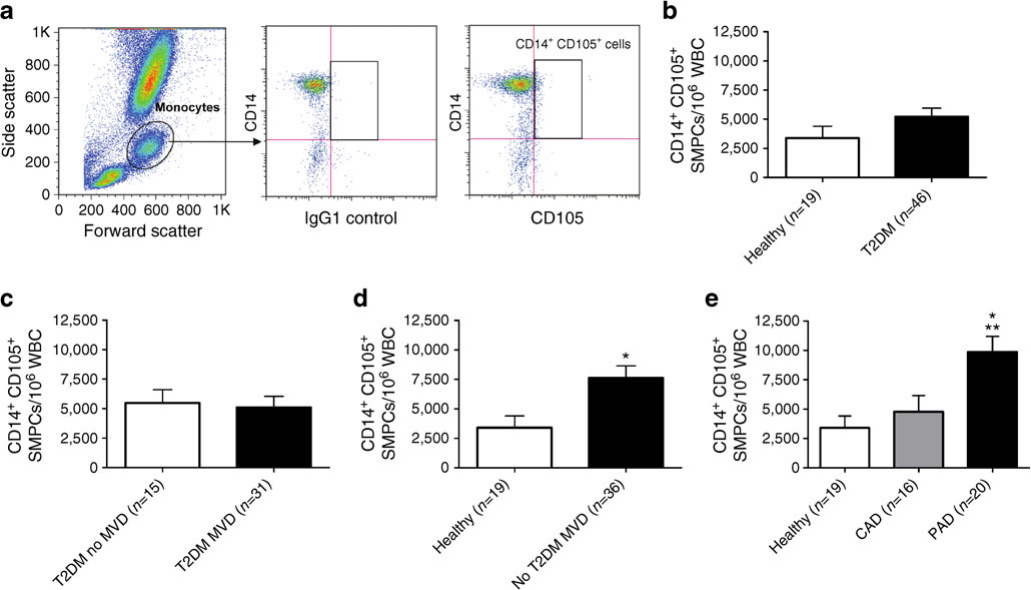
SMPC levels are increased in patients with MVD but without type 2 diabetes. (**a**) SMPCs were identified within a monocyte gate based on forward and side scatter characteristics. Within the monocyte gate, CD14^+^ cells expressing CD105 were quantified based on the isotype (IgG1) control. (**b**) SMPC levels were similar between type 2 diabetic patients and healthy controls. **c** Within type 2 diabetic patients there were no differences in the SMPC frequencies between patients with and without MVD. (**d**) Within non-diabetic patients, individuals with MVD had 2.2-fold higher circulating SMPC levels compared with healthy controls. (**e**) Only the presence of PAD was associated with increased numbers of SMPCs compared with healthy controls (*p* < 0.01) and CAD (*p* < 0.05). Data are expressed as mean values ± SEM; *p < 0.05 and **p < 0.01 T2DM, type 2 diabetes mellitus

In addition to EPCs, we also determined the levels of circulating CD14^+^CD105^+^ SMPCs in non-diabetic and diabetic individuals with and without MVD. As shown in Fig. 2a, CD14^+^CD105^+^ SMPCs represent a small but consistently identifiable subpopulation of CD14^+^ monocytes. When comparing healthy control individuals and diabetic patients (with and without MVD), no significant difference was observed (Fig. 2b). When subdividing the group of diabetic patients into individuals with and without MVD no difference between the two subgroups was observed (Fig. 2c). These results suggest that in diabetes there is a selective reduction in EPC levels, whereas SMPC levels are not significantly affected. However, in contrast to diabetic individuals, in non-diabetic individuals the number of circulating CD14^+^CD105^+^ SMPCs was significantly increased (2.2-fold) in participants with MVD when compared with healthy controls (Fig. 2d, *p* < 0.05). When subdividing the group of non-diabetic patients with MVD into patients with CAD or PAD, only PAD was associated with significantly increased SMPC levels (Fig. 2e, *p* < 0.05 and *p* < 0.01 vs CAD and healthy controls, respectively).

### Phenotypic characterisation of cultured CACs and SMPCs

**Fig. 3:**
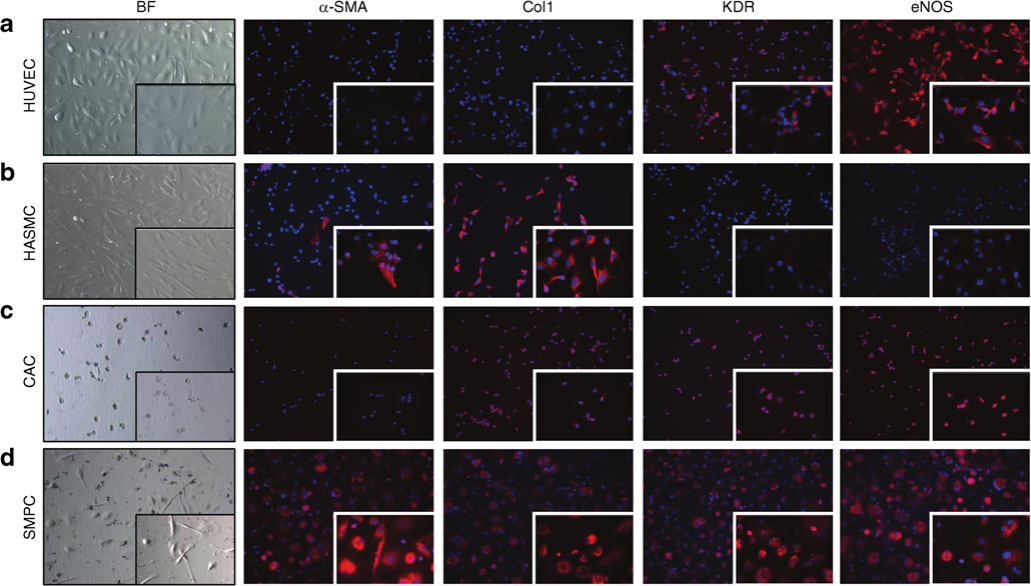
The phenotype of in vitro cultured HUVECs, HASMCs, CACs and SMPCs. Pictures were taken at × 200 and × 630 (inset) magnification. (**a**) HUVECs and (**b**) HASMCs were used as positive controls for the assessment of expression of EC and SMC differentiation markers. (**c**) CACs contained collagen type 1, KDR and eNOS, but not α-SMA. (**d**) SMPCs contained α-SMA, collagen type 1, KDR and eNOS. Nuclear staining is shown in blue (DAPI) while positive staining with the respective antibodies is shown in red. Col1, collagen type 1. BF, Bright field

Next we determined CAC and SMPC frequency in the peripheral blood as well as their respective differentiation capacities towards ECs and SMCs in vitro. HUVECs and human aortic smooth muscle cells (HASMCs) served as positive control cells for endothelial- and smooth-muscle-specific antibodies. HUVECs were positive for KDR and eNOS but negative for α-SMA and collagen type 1, whereas HASMCs were positive for α-SMA and collagen type 1 but did not contain KDR or eNOS (Fig. 3a, b). When PBMCs were cultured for 7 days under conditions favouring the outgrowth of CACs, adherent cells showed a small and rounded morphology. These cells were positive for KDR, eNOS and collagen type 1, but these in vitro cultured CACs did not contain α-SMA (Fig. 3c). In contrast, SMPCs contained α-SMA as well as KDR, eNOS and collagen type 1 (Fig. 3d), thereby clearly demonstrating phenotypic overlap of cultured CACs and SMPCs. SMPCs showed an elongated phenotype resembling HASMCs. We also performed immunofluorescence for the SMC differentiation markers calponin and smooth muscle myosin heavy chain. However, we found that both cultured CACs and SMPCs did not produce these proteins (data not shown). To analyse the phenotype of in vitro cultured CACs and SMPCs in more detail, real-time RT-PCR was performed for a selected set of EC and SMC lineage markers using a Taqman Low Density Array platform. We found that SMPCs derived from healthy controls expressed significantly higher levels of *α-SMA* (also known as *ACTA2*) and collagen type 1 mRNA when compared with EPCs from the same individuals. On the other hand, CACs expressed higher transcript levels of *CD31* (also known as *PECAM1*) and *eNOS* (also known as *NOS3*; data not shown). In line with our immunofluorescence results, we found overlap between the expression of several EC and SMC marker genes in both CACs and SMPCs (data not shown).

### Quantification of cultured CACs and SMPCs

**Fig. 4:**
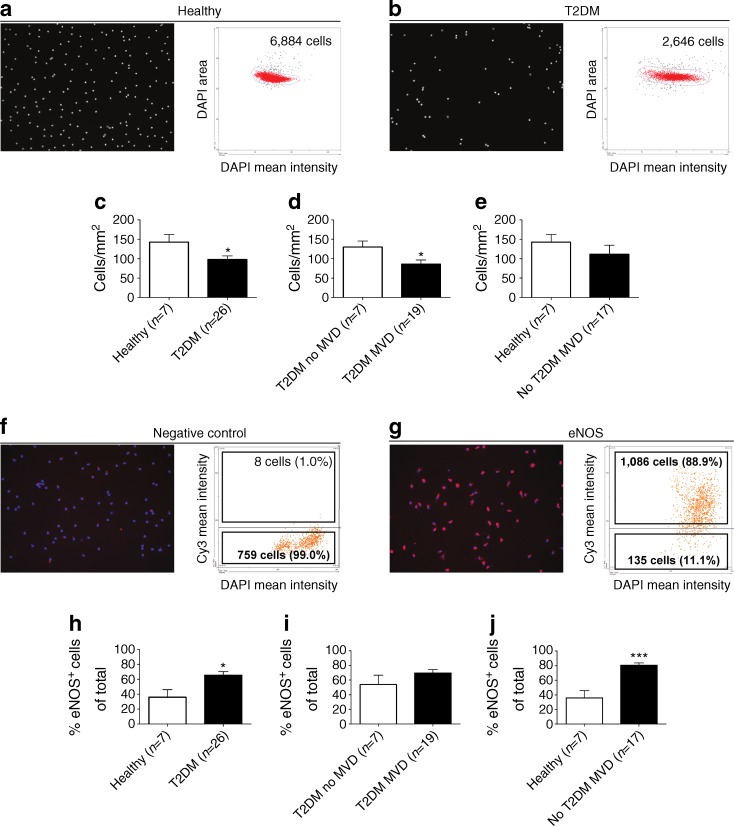
CAC outgrowth in culture is reduced in type 2 diabetic patients. (**a**,**b**) Representative images and corresponding scatterplots of DAPI-stained CAC nuclei, which were quantified with the TissueFAXS system. The scatterplots show the total number of nuclei that were quantified in a fixed region in a representative healthy control **(a)** and a type 2 diabetic patient without MVD **(b)**. **(c)** CAC outgrowth was reduced 1.5-fold in type 2 diabetic patients compared with healthy controls. **(d)** Within type 2 diabetic individuals, patients with MVD displayed a 1.5-fold reduction in the number of outgrowth CACs compared with type 2 diabetic patients without MVD. **(e)** Within non-type 2 diabetic individuals, similar CAC levels were observed in individuals with and without MVD. **(f,g)** The presence of eNOS was assessed using TissueFAXS analysis: **(f)** negative control staining; and **(g)** eNOS staining. **(h)** The percentage of CACs containing eNOS was increased in type 2 diabetic patients compared with healthy controls **(h)**, but not according to MVD status in type 2 diabetic patients **(i)**. **(j)** Individuals with MVD but not type 2 diabetes had significantly higher frequencies of CACs containing eNOS compared with healthy controls. Data are expressed as mean values ± SEM; *p < 0.05, ***p < 0.001. T2DM, type 2 diabetes mellitus

We hypothesised that diabetes and MVD are associated with differences in CAC and SMPC numbers in addition to an altered differentiation potential. To test this hypothesis we first quantified total CAC and SMPC outgrowth potential in vitro by analysing the number of CACs and SMPCs present after culture based on nuclear DAPI staining using the TissueFAXS system. To test if the differentiation potential of CACs and SMPCs is altered by the presence of diabetes and MVD we next quantified the number of cells positive for the EC and SMC markers described above, as well as the staining intensity of these markers as shown in Fig. 3. Figure 4a,b depicts a representative example of quantitative analyses of the total number of cultured CACs present from a healthy individual (Fig. 4a) and a diabetic patient without MVD (Fig. 4b) within a fixed region in a chamber slide well. Nuclei present on a total surface area of 29.1 mm^2^ were captured and quantitatively analysed using TissueQuest analysis software. As depicted in Fig. 4a, a sample derived from a healthy control had a higher CAC count compared with that from a diabetic patient without MVD. Quantitative analysis revealed that CAC outgrowth (expressed as cells per mm^2^) was significantly reduced (1.5-fold reduction) in diabetic patients (with or without MVD) compared with healthy controls (Fig. 4c, *p* < 0.05). When dividing diabetic individuals into patients with and without MVD, significantly reduced (1.5-fold reduction) CAC outgrowth was observed in diabetic patients with MVD compared with patients without MVD (Fig. 4d, *p* < 0.05). When we expressed the number of CACs per 10^6^ WBCs we also found a reduction in CAC frequency in diabetic patients with MVD compared with diabetic patients without MVD (ESM Table 1). This MVD-associated additional reduction in CAC outgrowth was not observed in non-diabetic individuals with MVD (Fig. 4e).

**Fig. 5:**
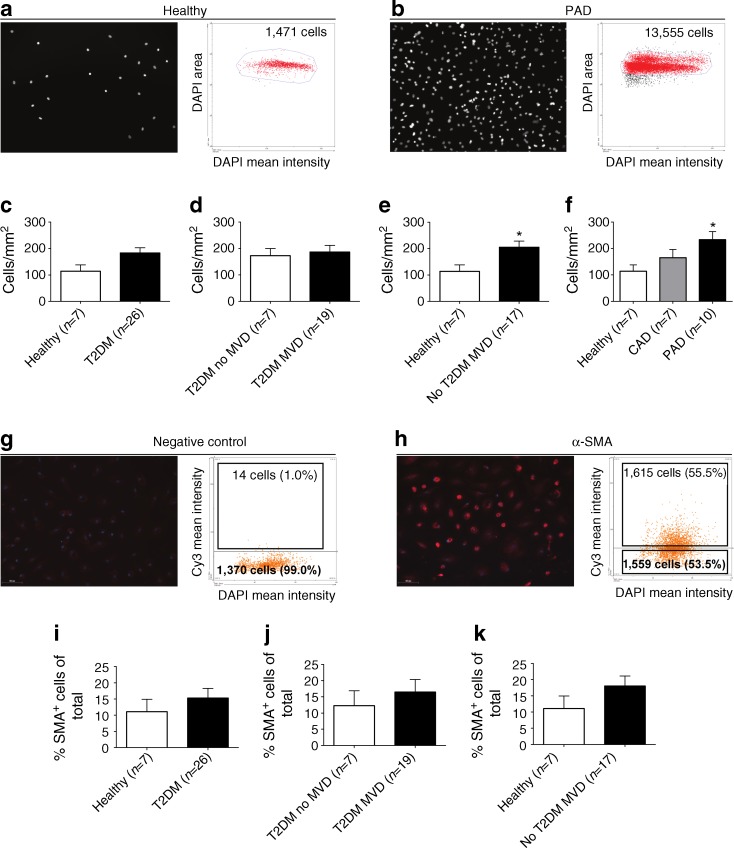
SMPC outgrowth is increased in non-type 2 diabetic patients with PAD. **(a,b)** Representative images and corresponding scatterplots of DAPI-stained SMPC nuclei quantified with the TissueFAXS system. The scatterplots show the total number of nuclei that were quantified in a fixed region in a representative healthy control **(a)** and a non-type 2 diabetic patient with PAD **(b)**. **(c)** There was no significant difference in SMPC numbers between type 2 diabetic patients and healthy controls. **(d)** Within type 2 diabetic patients, similar SMPC numbers were detected in patients with and without MVD. **(e)** Within non-diabetic individuals, patients with MVD had a 1.8-fold increase in the number of outgrowth SMPCs compared with healthy controls. **(f)** Within non-diabetic patients, SMPC levels were increased 2.0-fold in PAD patients compared with healthy controls. **(g,h)** The presence of α-SMA was assessed using TissueFAXS analysis: **(g)** negative control staining; and **(h)** α-SMA staining. There was a tendency towards increased percentages of α-SMA^+^ outgrowth SMPCs in: **(i)** type 2 diabetic patients with and without MVD compared with healthy controls; **(j)** type 2 diabetic patients with MVD, compared with those without MVD; and **(k)** non-type 2 diabetic patients with MVD compared with healthy controls. Data are expressed as mean values ± SEM; *p < 0.05. T2DM, type 2 diabetes mellitus

Quantification of SMPC outgrowth after in vitro culture was performed in a similar manner to that for cultured CACs as described above. Figure 5a,b depicts a representative example of quantitative analyses of the total number of cultured SMPCs from a healthy individual present within a fixed region in a chamber slide well (left two panels) and from a non-diabetic patient with PAD (right two panels). In line with our CD14^+^CD105^+^ FACS data (Fig. 2b) we did not find a significant difference in SMPC numbers after in vitro culture (expressed as cells per mm^2^) in diabetic patients when compared with healthy controls (Fig. 5c). However, when we expressed the number of SMPCs per 10^6^ WBCs or as number of cells per ml blood we did find significantly increased SMPC numbers in diabetic patients compared with healthy controls (ESM Table 1). No difference in SMPC outgrowth was observed between diabetic patients with and without MVD (Fig. 5d). However, in non-diabetic individuals a significantly higher number (1.8-fold increase) of outgrowth SMPCs were observed in patients with MVD compared with healthy controls (Fig. 5e, *p* < 0.05). When stratifying non-diabetic patients with MVD into patients with either PAD or CAD we observed that only the presence of PAD, but not CAD, was associated with significantly increased (twofold) SMPC outgrowth when compared with healthy controls (Fig. 5f, *p* < 0.05).

### Differentiation of cultured CACs and SMPCs

To assess whether the differentiation potential of outgrowth CACs towards an EC phenotype was altered in diabetes and MVD we determined the percentage of CACs positive for KDR or eNOS staining using TissueFAXS analysis. Figure 4f,g shows a representative example of the gating strategy performed using TissueQuest analysis software. The proportion of CACs producing KDR was similar among all groups. However, the percentage of CACs producing eNOS was significantly increased in diabetic patients (with or without MVD, Fig. 4h, *p* < 0.05) and non-diabetic patients with MVD (Fig. 4j, *p* < 0.001) compared with healthy controls. The percentage of eNOS^+^ CACs was similar between diabetic patients with and without MVD (Fig. 4i). Next, we compared the mean fluorescence intensity ratio of the eNOS staining to investigate if the average level of eNOS per cell was affected by diabetes or MVD. Despite the higher percentage of CACs producing eNOS in diabetic and non-diabetic patients with MVD, the eNOS staining intensity was similar among the groups (not shown). In addition, the absolute number of eNOS^+^ CACs per mm^2^ was not altered (not shown). Real-time RT-PCR showed that there was no difference in mRNA expression of EC differentiation markers among the groups (ESM Table 2). Taken together, the results from the TissueFAXS quantification and the differentiation analyses show that diabetes and MVD are associated with a reduction in the number of cultured CACs, but the differentiation capacity of these cells is unaltered.

As for CACs, we assessed whether diabetes or MVD affected the differentiation potential of outgrowth SMPCs. To this end we quantified the percentage of SMPCs containing α-SMA with immunofluorescence. A representative example of the gating strategy performed using TissueQuest analysis software is shown in Fig. 5g,h. Although the percentages of α-SMA^+^ outgrowth SMPCs tended to be higher in diabetic patients with and without MVD (vs healthy controls, Fig. 5i), in diabetic patients with MVD (vs diabetes without MVD, Fig. 5j), and in non-diabetic patients with MVD (vs healthy controls, Fig. 5k), no significant differences were observed because of relatively large variations within the groups. Gene expression data obtained with real-time RT-PCR showed that several genes were differentially expressed in SMPCs when comparing all groups (ESM Table 3). However, we did not observe an association of differential expression of a complete panel of EC or SMC marker genes with diabetes or MVD, indicating that the differentiation potential of SMPCs is not altered by either of these diseases.

### Type 2 diabetes is associated with reduced CAC/SMPC and EPC/SMPC ratios

**Fig. 6:**
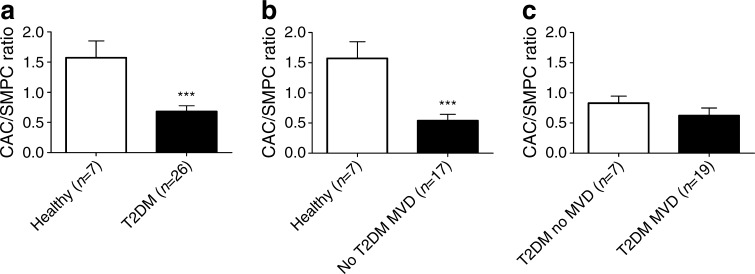
The CAC/SMPC ratio (cells per mm^2^) is decreased in type 2 diabetic patients and individuals with MVD. **(a)** The CAC/SMPC ratio shows a 2.3-fold decrease in type 2 diabetic patients compared with healthy controls. **(b)** Within non-diabetic individuals, the CAC/SMPC ratio was decreased 2.9-fold in patients with MVD compared with healthy controls. **(c)** Within patients with type 2 diabetes there was no difference in the CAC/SMPC ratio between patients with or without MVD. Data are expressed as mean values ± SEM; ***p < 0.001. T2DM, type 2 diabetes mellitus

To determine if the balance between CACs and SMPCs was disturbed by the presence of diabetes and/or MVD, the CAC/SMPC ratio was calculated based on the in vitro data shown in Figs 4 and 5. Compared with healthy controls, the CAC/SMPC ratio is significantly reduced (2.3-fold reduction) in diabetes (Fig. 6a, *p* < 0.001). In non-diabetic individuals, the presence of MVD was associated with a 2.9-fold reduction in the CAC/SMPC ratio (Fig. 6b, *p* < 0.001). As diabetes without MVD was already associated with a markedly reduced CAC/SMPC ratio, no additional reduction was observed in diabetes with MVD (Fig. 6c). When we calculated the EPC/SMPC ratio based on the data obtained with FACS we found a significantly reduced CD34^+^ cell/CD14^+^CD105^+^ SMPC ratio (2.7-fold reduction, *p* < 0.01) and CD34^+^KDR^+^ cell/CD14^+^CD105^+^ SMPC ratio (3.7-fold reduction, *p* < 0.01) in diabetic patients compared with healthy controls. There was no additional reduction of these ratios associated with MVD in diabetic or non-diabetic individuals (data not shown). Together these data indicate that the presence of type 2 diabetes is associated with a significant decrease in the CAC/SMPC and EPC/SMPC ratios.

## Discussion

The main finding of this study is that the numerical balance between circulating progenitor cells with vasoregenerative capacity (EPCs and CACs) and damaging SMPCs is altered in favour of SMPCs in type 2 diabetes. Using established FACS protocols for the detection of EPCs and SMPCs we observed that the frequency of circulating CD34^+^ and CD34^+^KDR^+^ cells is reduced in patients with diabetes, whereas the levels of CD14^+^CD105^+^ SMPCs are not significantly changed by the presence of diabetes. We additionally investigated CAC and SMPC frequency and phenotype using in vitro cell culture of patient-derived PBMCs under pro-angiogenic or pro-fibrotic conditions. Under these conditions a subset of adherent PBMCs differentiated towards CACs or SMPCs, respectively. Similar to the results obtained with FACS for EPCs, we found a significant reduction in CAC outgrowth in vitro when culturing PBMCs derived from diabetes patients. The presence of MVD (either CAD or PAD) in diabetes was associated with an additional decrease in CACs. This MVD-associated reduction in CAC levels in vitro was not observed in our FACS analysis of EPCs. A likely explanation for this discrepancy is that cultured CACs represent a subtype of vasoregenerative cells that is different from the bone-marrow-derived EPCs originally described by Asahara et al [4]. These different cell types may be differentially affected by MVD. It has been shown that CACs cultured in vitro are derived from a subset of CD14^+^ monocytes rather than CD34^+^ bone marrow-derived progenitor cells as identified by FACS [23]. SMPC outgrowth potential in vitro was not significantly affected by diabetes. This suggests that diabetes predominantly causes a decrease in the number of EPCs and CACs rather than an increase in SMPCs.

Diabetes is shown to cause defective progenitor cell mobilisation from the bone marrow in response to ischaemia [24]. However, it seems that this is limited to EPCs and CACs as we show that SMPC levels remain the same or are even slightly elevated in diabetic patients, regardless of the presence of MVD. These results contrast with the data reported by Nguyen et al showing significantly increased SMPC levels (referred to as myofibroblast progenitor cells [MFPCs] in their study) in type 1 diabetes patients that could possibly influence MVD rates in these patients [22]. These contrasting observations cannot be explained by differences in glycaemic control between the study populations because in both studies participants were relatively well controlled as judged by HbA_1c_ levels. Our results are in agreement with a study performed by Westerweel et al who found reduced EPC numbers with preserved SMPC levels in patients with ESRD compared with healthy controls [21]. As for diabetic patients, individuals with ESRD are at increased cardiovascular risk including MVD, underscoring the association of disturbed EPC/SMPC and CAC/SMPC balances with adverse vascular remodelling.

The ratio between EPC or CAC numbers and SMPC numbers was significantly lower for individuals with diabetes compared with healthy controls. This reduced ratio was independent of the presence of MVD, suggesting also that diabetic patients without MVD are at increased risk of developing MVD, despite not having the clinical symptoms at the time of analysis.

In contrast to diabetic individuals with MVD, the presence of MVD in non-diabetic patients was associated with significantly increased SMPC levels. This increase was confined to non-diabetic patients with PAD, as demonstrated with both FACS analysis and in vitro cell culture. Increased SMPC levels were not found in non-diabetic patients with CAD despite similar baseline characteristics and metabolic variables. Our data therefore suggest that the imbalance in EPC/CAC and SMPC levels is more relevant for the development of PAD than CAD in non-diabetic patients. The mechanism behind this observation remains to be identified.

In addition to numerical changes in EPC, CAC and SMPC frequency in diabetic patients, we also investigated the differentiation capacities of CACs and SMPCs in vitro. An altered differentiation capacity of progenitor cells might contribute to increased rates of atherosclerosis in diabetic patients. Increased differentiation of progenitor cells towards SMCs could promote plaque growth by increasing the volume of the fibrous cap. On the other hand, reduced differentiation of CACs towards mature ECs could inhibit arteriogenesis and angiogenesis, which could worsen ischaemia downstream of the plaque. However, using EC- and SMC-lineage-specific immunofluorescence and gene expression analysis we found that the phenotype of outgrowth CACs and SMPCs in vitro was similar among the groups. Therefore, numerical changes rather than an altered differentiation capacity of CACs and SMPCs may contribute to the development of MVD.

In conclusion, our study demonstrates a disturbed balance between circulating EPCs or CACs and SMPCs in type 2 diabetes in favour of a numerical decrease in EPCs and CACs, resulting in a relative increase in SMPC numbers. This imbalance may contribute to a decreased capacity for vascular repair in type 2 diabetic patients, increasing their risk of developing MVD.

## Electronic supplementary materials

Below is the link to the electronic supplementary material.

ESM Methods PDF 71.8 kb

ESM Table 1 PDF 11 kb

ESM Table 2 PDF 56 kb

ESM Table 3 PDF 58 kb

## Abbreviations

α-SMA: α-Smooth muscle actin
CAC: Circulating angiogenic cell
CAD: Coronary artery disease
EC: Endothelial cell
eNOS: Endothelial nitric oxide synthase
EPC: Endothelial progenitor cell
ESRD: End-stage renal disease
HASMC: Human aortic smooth muscle cell
KDR: Kinase domain receptor
MVD: Macrovascular disease
PAD: Peripheral artery disease
PBMC: Peripheral blood mononuclear cell
SMC: Smooth muscle cell
SMPC: Smooth muscle progenitor cell
WBC: White blood cell
