# Supplementary material for: Type 2 diabetes mellitus is associated with an imbalance in circulating endothelial and smooth muscle progenitor cell numbers
Source: Diabetologia. 2012 Jun 1;55(9):2501–12. doi: 10.1007/s00125-012-2590-5 (PMC3411291; doi:10.1007/s00125-012-2590-5)
Supplement: Supplementary file 1 — PDF 71.8 kb [file 125_2012_2590_MOESM1_ESM.pdf]

Electronic supplementary material

Materials and Methods

1.1 Subjects

PAD of the lower extremities was diagnosed on the basis of a history of claudication or rest pain, bilateral pulses examination and duplex ultrasound. Patients were graded according to the Leriche/Fontaine clinical classification of chronic lower extremity ischemia [1]. Diagnosis of CAD was based on prior myocardial infarction (>6 months), or of evidence of significant coronary artery stenosis during angiography. Patients with clinical evidence of both CAD and PAD were excluded from the study. Predefined exclusion criteria for all subjects were the following medical conditions: advanced microvascular disease (diabetic retinopathy and nephropathy), auto-immune diseases, neoplasm, acute or chronic infections, recent (<6 months) surgery or vascular intervention, age >80 years, recent (<6 months) myocardial infarction, haemodialysis and use of immunosuppressive medication. All subjects were screened for cardiovascular risk factors to establish confounding effects, including: smoking, hypertension, BMI and dyslipidaemia. Metabolic evaluation of all participants was performed including glucose, HbA_{1c}, lipid profile and serum creatinine.

1.2 Flow cytometry

Whole blood samples were centrifuged and plasma was aspirated. The pellet was resuspended in phosphate buffered saline (PBS) containing 5% fetal bovine serum (FBS), centrifuged and the supernatant discarded. This was repeated once more to remove remaining plasma components. After the last centrifugation step, 700 µL concentrated blood was incubated with FcR blocking reagent (Miltenyi Biotech GmbH, Bergisch Gladbach, Germany) for 10 minutes at 4ºC. To identify EPCs, 250 µL concentrated blood was incubated with PerCP-conjugated mouse-α-human CD34 (BD Biosciences) and FITC-conjugated mouse-α-human KDR (R&D Systems, McKinley Place, USA) antibodies at appropriate dilutions. To identify SMPCs, 50 µL blood was incubated with FITC-conjugated mouse-α-human CD105 (Biolegend, Roselle Street, USA) and PerCP-conjugated mouse-α-human CD14 (BD Biosciences, Franklin Lakes, NJ) antibodies at appropriate dilutions. Samples were incubated for 30 minutes at 4ºC after which erythrocytes were lysed in FACS lysing solution (BD Biosciences). Samples were then washed in PBS/ 5% FBS, fixed in 2% paraformaldehyde and stored overnight at 4ºC. Samples were analyzed within 24 hours on a FACSCalibur (BD Biosciences). Because EPCs are relatively rare in the peripheral blood $5x10^5$ events/sample were acquired to determine their frequency. For SMPCs $1x10^5$ events/sample were acquired. Analysis of FACS data was performed with Flowjo software (Tree Star Inc., USA). Samples stained with appropriate isotype-control antibodies (for EPCs: mouse IgG_1-FITC and mouse IgG_1-PerCP [Biolegend]; for SMPCs: mouse IgG_1-FITC [R&D Sytems] and mouse IgG_{2b}-PerCP [Biolegend]) served as negative controls and were used for gate setting. EPCs were identified within the white blood cell (WBC) gate as cells with a low side scatter expressing CD34. Within this $CD34^+$ population, the frequency of $CD34^+KDR^+$ cells was assessed. SMPCs resided within the monocyte gate and were phenotypically identified based on co-expression of CD14 and CD105.

1.3 CAC and SMPC culture

Peripheral blood mononuclear cells (PBMC) were isolated from blood using Ficoll density gradient centrifugation (Ficoll-Paque Plus, GE Healthcare, Buckinghamshire, UK). For quantification and characterization with immunofluorescence, CACs and SMPCs were cultured on fibronectin-coated

8-well permanox chamber slides (Nalge Nunc, Creek Drive, USA). For CAC culture, PBMCs were seeded at a density of $2x10^5$ cells/well in complete Endothelial Cell Growth Medium-2 (EGM-2, Promocell, Heidelberg, Germany) supplemented with antibiotics (Penicillin/Streptomycin, (Lonza, Verviers, Belgium). Medium was replenished at day 3 to remove non-adherent cells. At day 7 cells were washed once in PBS, then fixed in 90% acetone (10 minutes at -20 ºC), airdried and stored at -20 ºC for subsequent analysis. For SMPC culture, PMBCs were seeded at a density of $1x10^5$ cells/well in DMEM medium (1 g/L glucose) supplemented with 2 mM L-glutamine, 20% heat-inactivated FBS and antibiotics. Medium was replenished on day 3 and subsequently every 48-72 hours. After 21 days of culture cells were washed, fixed in 90% acetone and stored at -20ºC for subsequent analysis as described above. For gene expression analysis, CACs and SMPCs were cultured on fibronectin-coated 6-wells plates at a density of $1x10^7$ cells/well in their respective culture medium. Medium was replenished at day 3, and at day 7 cells were washed once in PBS and subsequently lysed in 350 µL RLT buffer (Qiagen, Venlo, the Netherlands) containing 1% v/v β-mercaptoethanol and stored at -80 ºC.

1.4 Characterization of cultured progenitor cells

To investigate the expression of EC and SMC differentiation markers by *in vitro* cultured CACs and SMPCs, immunofluorescent stainings were performed. To this end, frozen fixed cells were thawed, rehydrated in PBS, permeabilized in PBS/0.05% Triton for 3 minutes after which cells were incubated with primary antibodies diluted in PBS/1% BSA for 60 minutes at room temperature. Primary antibodies directed against the following antigens were used: α-smooth muscle actin (α–SMA, Clone 1A4, DAKO, Glostrup, Denmark), KDR (A-3, Santa Cruz, USA), endothelial nitric oxide synthase (eNOS, rabbit polyclonal, BD Biosciences), and Collagen Type 1 (goat polyclonal, Southern Biotech, Birmingham, USA). Subsequently, cells were incubated with appropriate biotinylated secondary antibodies for 30 minutes at room temperature. Next, cells were incubated with Alexa Fluor 555-conjugated streptavidin (Invitrogen, Carlsbad, USA) for 30 minutes at room temperature followed by 4',6-diamidino-2-phenylindole (DAPI, Invitrogen) nuclear counterstaining and mounting in Aqua Poly/Mount (Polysciences, Warrington, USA). To confirm the specificity of the primary antibodies for the EC and SMC lineage markers we performed immunofluorescent stainings on cultured human aortic smooth muscle cells (HASMC, ScienCell, Carlsbad, USA) and human umbilical vein endothelial cells (HUVEC, Endothelial Cell Facility UMCG, Groningen, The Netherlands), respectively.

1.5 Quantitative immunofluorescence: TissueFAXS analysis

Immunofluorescent stainings of *in vitro* cultured CACs and SMPCs were analyzed with the TissueFAXS system (Tissuegnostics, Vienna, Austria) with a Zeiss AxioObserver Z1. First, a preview of the whole chamberslide was acquired with a 2.5x objective using the DAPI filter to identify nuclei. Based on the preview, a region of 7x7 fields of view (FOV) was set for each well. Next, the regions were acquired with a 20x objective using a Cy3 and DAPI filter. The acquired regions were analyzed with TissueQuest software (version 3.0.1120.0137, Tissuegnostics, Vienna, Austria). Nuclei were identified based on the DAPI staining and gated to exclude cell debris. The mean intensity of Cy3 staining of nucleated cells was plotted against the DAPI mean intensity and a threshold was set based on the wells in which primary antibody was omitted (negative control). Cells with a staining intensity above this threshold were considered positive for binding of the antibody of interest. Data are expressed as absolute number of cells per mm^2 surface area as well as percentage of positive cells relative to the total number of cells in a fixed surface area. To quantify the relative expression level for each marker the mean fluorescence of intensity ratio (MFIR) was calculated by dividing the mean fluorescence intensity (MFI) of cells stained with specific antibodies by the MFI of negative controls.

1.6 Gene expression profile of in vitro cultured CACs and SMPCs

RNA was isolated from CACs and SMPCs (cultured for 7 days) using the RNeasy Micro kit (Qiagen, Venlo, the Netherlands) according to the manufacturers instructions. The quantity of RNA was measured with a Nanodrop spectrophotometer. For cDNA synthesis 300-1000 ng RNA was converted to cDNA using SuperScript II reverse transcriptase and random hexamere primers (Invitrogen) following the manufacturers instructions. mRNA expression of genes involved in EC and SMC differentiation (including 3 housekeeping genes; B2M, GAPDH and HPRT) was measured with Taqman Low Density Array (Applied Biosystems, Carlsbad, USA) using exon junction spanning primers. Reactions were performed on a Taqman 7900HT Real-Time PCR System following the manufacturer's instructions. Gene expression was analyzed using SDS 2.2.2 Software. When we compared the gene expression of the 3 housekeeping genes we found all 3 showed consistent expression across all groups. We chose GAPDH to compare the gene expression levels. To obtain the ΔCt, the GAPDH Ct values were subtracted from the Ct value from each gene. We used the comparative Ct method ($2^{-\Delta Ct}$ method) to calculate the relative gene expression to the expression of GAPDH. To compare the expression levels between the patient groups we calculated the gene expression relative to the healthy control group by normalizing the $2^{-\Delta Ct}$ values to the healthy control group, which was set at 1.0

References

[1] Pentecost MJ, Criqui MH, Dorros G, et al. (2003) Guidelines for peripheral percutaneous transluminal angioplasty of the abdominal aorta and lower extremity vessels. A statement for health professionals from a Special Writing Group of the Councils on Cardiovascular Radiology, Arteriosclerosis, Cardio-Thoracic and Vascular Surgery, Clinical Cardiology, and Epidemiology and Prevention, the American Heart Association. J Vasc Interv Radiol 14:S495-515
