# Supplementary material for: Type 2 diabetes mellitus is associated with an imbalance in circulating endothelial and smooth muscle progenitor cell numbers
Source: Diabetologia. 2012 Jun 1;55(9):2501–12. doi: 10.1007/s00125-012-2590-5 (PMC3411291; doi:10.1007/s00125-012-2590-5)
Supplement: Supplementary file 2 — PDF 11 kb [file 125_2012_2590_MOESM2_ESM.pdf]

ESM Table 1

Table 1. CAC and SMPC levels *in vitro* expressed as 10^3 cells per 10^6 WBC and per mL blood.

		Non-Type 2 diabetes					Type 2 diabetes				
		**all**	**no MVD**	**MVD**	**PAD**	**CAD**	**all**	**no MVD**	**MVD**	**PAD**	**CAD**
	n	24	7	17	10	7	26	7	19	10	9
**CAC**	$x10^3$ per 10^6 WBC	10.5 ± 1.4	11.3 ± 2.0	10.2 ± 1.8	13.3 ± 2.6	5.7 ± 1.5	8.3 ± 0.9	11.2 ± 1.9[a]	7.0 ± 0.9	5.9 ± 0.9	8.6 ± 1.6
	$x10^3$ per mL blood	77.8 ± 14.1	60.3 ± 11.9	84.9 ± 19.3	120.0 ± 27.8[b]	35.0 ± 7.2	61.0 ± 6.2	78.6 ± 13.2	54.6 ± 6.6	49.7 ± 9.2	55.7 ± 10.6
**SMPC**	$x10^3$ per 10^6 WBC	33.3 ± 4.2	17.2 ± 3.3[c]	39.9 ± 5.0	44.7 ± 6.9	33.0 ± 6.8	29.9 ± 2.7	29.3 ± 4.9	30.2 ± 3.3	32.2 ± 4.9	28.0 ± 4.6
	$x10^3$ per mL blood	250.0 ± 44.1	94.1 ± 20.8[d]	318.8 ± 57.2	407.8 ± 83.4	191.6 ± 41.5	232.2 ± 32.0	235.3 ± 85.7	231.2 ± 33.9	275.8 ± 56.7	181.7 ± 29.7

Data presented as mean ± SEM.

Statistically significant with ANOVA compared to:
[a]T2DM no MVD compared to T2DM with MVD (p<0.05)
[b]Non-T2DM with PAD compared to non-T2DM with CAD (p<0.05)
[c]Healthy compared with T2DM, non-T2DM with MVD and non-T2DM with PAD (p<0.05).
[d]Healthy compared with T2DM (p<0.05), non-T2DM with MVD (p<0.05) and non-T2DM with PAD (p<0.01)
