# Supplementary material for: Type 2 diabetes mellitus is associated with an imbalance in circulating endothelial and smooth muscle progenitor cell numbers
Source: Diabetologia. 2012 Jun 1;55(9):2501–12. doi: 10.1007/s00125-012-2590-5 (PMC3411291; doi:10.1007/s00125-012-2590-5)
Supplement: Supplementary file 3 — PDF 56 kb [file 125_2012_2590_MOESM3_ESM.pdf]

ESM Table 2

Table 2. Gene expression of SMC and EC lineage genes in cultured CACs.

Gene	Type 2 diabetes without MVD	Type 2 diabetes with PAD	Tye 2 diabetes with CAD	Healthy control	Non-type 2 diabetes with PAD	Non-type 2 diabetes with CAD	p-value
**SMC**							
ACTA2	1.4 ± 0.4	0.8 ± 0.1 Δ	0.6 ± 0.1 Δ	1.0 ± 0.3	0.4 ± 0.2	0.7 ± 0.2 Δ	NS
SMT	2.0 ± 0.6	1.9 ± 0.6	1.6 ± 0.4	1.0 ± 0.2	1.1 ± 0.3	1.6 ± 0.3	NS
SRF	1.1 ± 0.2	1.0 ± 0.1	1.0 ± 0.6	1.0 ± 0.1	0.8 ± 0.1	1.1 ± 0.2	NS
KLF4	1.4 ± 0.4	1.2 ± 0.2	1.7 ± 0.6	1.0 ± 0.2	0.9 ± 0.2	0.9 ± 0.2	NS
SMAD4	1.5 ± 0.2	1.1 ± 0.1	1.1 ± 0.1	1.0 ± 0.04	0.9 ± 0.04 [a]	1.0 ± 0.03	<0.05
TGFB1	1.3 ± 0.2	1.3 ± 0.1	1.7 ± 0.2	1.0 ± 0.1	1.0 ± 0.1	1.1 ± 0.04 Δ	NS
TGFBR1	1.5 ± 0.4	1.1 ± 0.1	1.1 ± 0.2	1.0 ± 0.1	1.0 ± 0.1	1.1 ± 0.2	NS
TGFBR2	1.3 ± 0.2	0.9 ± 0.1	1.0 ± 0.1	1.0 ± 0.1	0.8 ± 0.1	1.1 ± 0.1	NS
BMP4	1.1 ± 0.4	0.5 ± 0.1	0.3 ± 0.1	1.0 ± 0.3	0.4 ± 0.1	0.5 ± 0.1	NS
BMP6	3.4 ± 1.3	5.9 ± 1.4[b]	0.7 ± 0.4 Δ	1.0 ± 0.4	1.3 ± 0.4	4.0 ± 1.8	<0.05
PDGFBB	1.7 ± 0.3	1.4 ± 0.3	1.0 ± 0.3	1.0 ± 0.2	1.2 ± 0.1	1.2 ± 0.2	NS
**EC**							
KDR	0.5 ± 0.2 Δ	0.2± 0.1	0.5 ± 0.2	1.0 ± 0.4	0.3 ± 0.1	0.3 ± 0.2	NS
VEGFA	0.9 ± 0.2 Δ	1.1 ± 0.1	1.3 ± 0.2	1.0 ± 0.2	1.2 ± 0.3	1.5 ± 0.4	NS
NOS3	0.8 ± 0.3 Δ	1.4 ± 0.5	0.4 ± 0.1 Δ	1.0 ± 0.3	0.8 ± 0.2 Δ	1.1 ± 0.2	NS
CDH5	2.9 ± 1.0	8.3 ± 3.6	1.5 ± 0.6 Δ	1.0 ± 0.5	1.6 ± 0.4	1.4± 0.7	NS
PECAM1	2.0 ± 0.7	1.0 ± 0.1	1.0 ± 0.1	1.0 ± 0.1	0.9 ± 0.1	1.1 ± 0.1	NS

Data are expressed as relative mRNA expression +/- SEM compared to the healthy control group. N=6 per group unless indicated by Δ (N=5). *ACTA2*, α-Smooth muscle actin; *SMT*, Smoothelin, *SRF*, Serum response factor; *KLF4*, Krüppel-like factor 4; *SMAD4*; Mothers against decapentaplegic homolog 4; *TGFB1*, Transforming growth factor-β; *TGFBR1*, Transforming growth factor-β receptor 1; *TGFBR2*, Transforming growth factor-β receptor 2; *BMP4*, Bone-morphogenetic protein 4; *BMP6*, Bone-morphogenetic protein 6; *PDGFBB*, Platelet-derived growth factor-bb; *KDR*, Kinase domain receptor; *VEGFA*, Vascular endothelial growth factor A; *NOS3*, Endothelial nitric oxide synthase; *CDH5, Vascular endothelial cadherin; PECAM1*; Platelet endothelial cell adhesion molecule 1

Statistically significant with ANOVA compared to:
[a]non-T2DM with PAD vs. non-T2DM with CAD
[b]T2DM with PAD vs. T2DM with CAD.
