# Supplementary material for: Type 2 diabetes mellitus is associated with an imbalance in circulating endothelial and smooth muscle progenitor cell numbers
Source: Diabetologia. 2012 Jun 1;55(9):2501–12. doi: 10.1007/s00125-012-2590-5 (PMC3411291; doi:10.1007/s00125-012-2590-5)
Supplement: Supplementary file 4 — PDF 58 kb [file 125_2012_2590_MOESM4_ESM.pdf]

ESM Table 3

Table 3. Gene expression of SMC and EC lineage genes in cultured SMPCs.

Gene name	Type 2 diabetes without MVD	Type 2 diabetes with PAD	Type 2 diabetes with CAD	Healthy control	Non-type 2 diabetes with PAD	Non-Type 2 diabetes with CAD	p-value
**SMC**							
ACTA2	1.0 ± 0.1[a]	0.9 ± 0.2	0.4 ± 0.1 Δ	1.0 ± 0.2 Δ	0.6 ± 0.1	0.9 ± 0.3	<0.05
MYH11	2.7 ± 1.0	0.8 ± 0.2	0.7 ± 0.2 Δ	1.0 ± 0.5 Δ	0.6 ± 0.2 Δ	1.3 ± 0.4 Δ	NS
SMT	1.5 ± 0.3	1.0 ± 0.1	1.2 ± 0.3	1.0 ± 0.2 Δ	1.4 ± 0.3	0.9 ± 0.2	NS
SRF	1.2 ± 0.2	1.0 ± 0.01 Δ	1.2 ± 0.1 Δ	1.0 ± 0.3	1.3 ± 0.7	1.3 ± 0.2	NS
KLF4	0.8 ± 0.1	0.6 ± 0.1	0.7 ± 0.1 Δ	1.0 ± 0.2	0.5± 0.1 Δ	1.0 ± 0.2	NS
SMAD4	1.0 ± 0.1	0.8 ± 0.1	0.9 ± 0.02 Δ	1.0 ± 0.2 Δ	0.8 ± 0.05 Δ	0.8 ± 0.03	NS
TGFB1	0.8 ± 0.1	0.9 ± 0.1	1.1 ± 0.1	1.0 ± 0.2 Δ	0.8 ± 0.1 Δ	0.8 ± 0.1	NS
TGFBR1	0.6 ± 0.1	0.6 ± 0.05 Δ[b]	0.9 ± 0.1 Δ	1.0 ± 0.2 Δ	0.9 ± 0.2	1.0 ± 0.2	<0.05
TGFBR2	0.5 ± 0.1[c]	0.6 ± 0.03	0.8 ± 0.03 Δ[d]	1.0 ± 0.2	0.8 ± 0.1	0.6 ± 0.1	<0.05
BMP6	1.0 ± 0.3 Δ	1.9 ± 0.4[e]	0.7 ± 0.8	1.0 ± 0.2 Δ[f]	1.0 ± 0.2	0.4 ± 0.1 Δ	<0.05
PDGFBB	0.6 ± 0.1 Δ	1.2 ± 0.3	1.2 ± 0.2 Δ	1.0 ± 0.2 Δ	1.5 ± 0.3[g]	0.8 ± 0.1	<0.05
COL1A1	0.5 ± 0.2	0.3 ± 0.1 Δ	0.9 ± 0.3	1.0 ± 0.3	0.4 ± 0.2	0.8 ± 0.3	NS
**EC**							
KDR	0.3 ± 0.1[h]	1.5 ± 0.4	0.8 ± 0.2	1.0 ± 0.4	1.4 ± 0.5	1.1 ± 0.5	<0.05
VEGFA	1.2 ± 0.2[i]	0.7 ± 0.1	1.0 ± 0.1	1.0 ± 0.2 Δ	0.8 ± 0.2	1.4 ± 0.3	<0.05
NOS3	0.5 ± 0.1[j]	0.3 ± 0.05	0.5 ± 0.1 Δ	1.0 ± 0.1 Δ	0.7 ± 0.3	0.7 ± 0.2	<0.05
PECAM1	0.5 ± 0.1 Δ[k]	0.8 ± 0.1[l]	1.0 ± 0.04 Δ[m]	1.0 ± 0.2 Δ	0.8 ± 0.2	1.0 ± 0.2	<0.05

Data are expressed as relative mRNA expression +/- SEM compared to the healthy control group. N=6 per group unless indicated by Δ (N=5). *ACTA2*, α-Smooth muscle actin; *MYH11*, Smooth muscle myosin heavy chain; *SMT*, Smoothelin, *SRF*, Serum response factor; *KLF4*, Krüppel-like factor 4; *SMAD4*; Mothers against decapentaplegic homolog 4; *TGFB1*, Transforming growth factor-β; *TGFBR1*, Transforming growth factor-β receptor 1; *TGFBR2*, Transforming growth factor-β receptor 2; *BMP6*, Bone-morphogenetic protein 6; *PDGFBB*, Platelet-derived growth factor-bb; *COL1A1*; Collagen type 1, α1; *KDR*, Kinase domain receptor; *VEGFA*, Vascular endothelial growth factor A; *NOS3*, Endothelial nitric oxide synthase; *PECAM1*; Platelet endothelial cell adhesion molecule 1

Statistically significant with ANOVA compared to:

[a]T2DM no MVD vs. T2DM with CAD
[b] T2DM with PAD vs. T2DM with CAD
[c]T2DM no MVD vs. Healthy control
[d]T2DM no MVD vs. T2DM with CAD
[e]T2DM with PAD vs. T2DM with CAD
[f]Healthy control vs. non-T2DM with CAD
[g]non-T2DM with PAD vs. non-T2DM with CAD
[h]T2DM no MVD vs. T2DM with PAD
[i]T2DM no MVD vs. T2DM with PAD
[j]T2DM no MVD vs. Healthy control
[k]T2DM no MVD vs. Healthy control
[l]T2DM no MVD vs. T2DM with CAD
[m]T2DM with CAD vs. T2DM with PAD
